# *Lactobacillus rhamnosus* lowers zebrafish lipid content by changing gut microbiota and host transcription of genes involved in lipid metabolism

**DOI:** 10.1038/srep09336

**Published:** 2015-03-30

**Authors:** Silvia Falcinelli, Simona Picchietti, Ana Rodiles, Lina Cossignani, Daniel L. Merrifield, Anna Rita Taddei, Francesca Maradonna, Ike Olivotto, Giorgia Gioacchini, Oliana Carnevali

**Affiliations:** 1 Dipartimento di Scienze della Vita e dell'Ambiente, Università Politecnica delle Marche, Ancona, Italy; 2 Department for Innovation in Biological, Agro-food and Forest Systems (DIBAF), University of Tuscia, Viterbo, Italy; 3 Aquatic Animal Nutrition and Health Research Group, School of Biological Sciences, Plymouth University, PL4 8AA, UK; 4 Dipartimento di Scienze Economico-Estimative e degli Alimenti, Sezione di Chimica Bromatologica, Biochimica, Fisiologia e Nutrizione, Università degli Studi di Perugia, Perugia, Italy; 5 Section of Electron Microscopy, Great Equipment Center. Tuscia University, 01100 Viterbo, Italy

## Abstract

The microbiome plays an important role in lipid metabolism but how the introduction of probiotic communities affects host lipid metabolism is poorly understood. Using a multidisciplinary approach we addressed this knowledge gap using the zebrafish model by coupling high-throughput sequencing with biochemical, molecular and morphological analysis to evaluate the changes in the intestine. Analysis of bacterial 16S libraries revealed that *Lactobacillus rhamnosus* was able to modulate the gut microbiome of zebrafish larvae, elevating the abundance of Firmicutes sequences and reducing the abundance of Actinobacteria. The gut microbiome changes modulated host lipid processing by inducing transcriptional down-regulation of genes involved in cholesterol and triglycerides metabolism (*fit2, agpat4, dgat2, mgll*, *hnf4α, scap*, and *cck*) concomitantly decreasing total body cholesterol and triglyceride content and increasing fatty acid levels. *L. rhamnosus* treatment also increased microvilli and enterocyte lengths and decreased lipid droplet size in the intestinal epithelium. These changes resulted in elevated zebrafish larval growth. This integrated system investigation demonstrates probiotic modulation of the gut microbiome, highlights a novel gene network involved in lipid metabolism, provides an insight into how the microbiome regulates molecules involved in lipid metabolism, and reveals a new potential role for *L. rhamnosus* in the treatment of lipid disorders.

Probiotics positively modulate the gut microbiota, and recent studies revealed that host microbiota can modify host nutrient metabolism and energy balance both in germ free mice and zebrafish^1^,^2^. The gut microbiota has been considered as one of the most important factors influencing host metabolism, energy and lipid metabolism, fat distribution, insulin sensitivity and growth performance^3^.

Considering the increasing number of diseases associated with lipid metabolic disturbances, several studies have focused on the ability of probiotics, mainly *Lactobacillus* and *Bifidobacterium*, to decrease serum lipid content (*e.g.* cholesterol and triglycerides)^1^,^3^. Numerous hypotheses have been proposed for the mechanisms involved in the reduction of cholesterol by probiotics in the gut and their impact on the energy of the host^4^,^5^.

In general, the lipid metabolic pathway involves a considerable number of genes which regulate lipid synthesis, traffic, storage and homeostasis. Fish and mammals use triglycerides (TAG) and cholesterol derived from the diet as the main source of energy^6^. In eukaryotes, both the glycerol phosphate and the monoacylglycerol pathways represent the two main processes to resynthesize TAG^7^. TAG synthesis via the glycerol phosphate pathway mainly takes place in the liver and adipose tissues^8^. Briefly, this process involves the acylation of glycerol 3-phosphate by glycerol-3-phosphate acyltransferase (GAPT) and the conversion of diacylglycerol (DAG) into TAG by diacylglycerol acyltransferase (DGAT)^8^,^9^,^10^.

In order for the intestine to absorb TAG, TAG must first be hydrolyzed trough a three-step process catalyzed by adipose triglyceride lipase, hormonesensitive lipase, and monoacylglycerol lipase (MGLL)^11^,^12^. In particular, recent studies showed that MGLL is located in villi, enterocytes and adipocytes and is involved in the final step of TAG hydrolysis, specifically, hydrolyzing monoglycerides to free glycerol and fatty acids^13^,^14^,^15^. MGLL is also involved in the regulation 2-arachidonoylglycerol signalling^16^. Free glycerol and fatty acids are then taken up by the enterocytes, resynthesized into new TAG and either incorporated into nascent chylomicrons for secretion or stored as lipid droplets (LDs)^16^,^17^.

The monoacylglycerol pathway takes place in the intestine; the biosynthesis of TAG starts with the formation of bonds between monoacylglycerol and fatty acyl–CoA, catalyzed by monoacylglycerol acyltransferase, forming DAG^18^. Later, DGAT catalyzes a reaction that produces TAG from DAG and fatty acyl-CoA^18^. TAG together with sterol esters and phospholipids form LDs, which are cytosolic structures located in numerous cell types including enterocytes, which play a crucial role in the energy homeostasis in all eukaryotes^19^.

On the other hand, free cholesterol from biliary or dietary origin is absorbed by the enterocytes and incorporated into chylomicrons as cholesterol ester (CE), then transported into the lymphatic system^6^.

The present study aimed at evaluating the effect of supplementation of the probiotic *L. rhamnosus* on the gut microbial community and lipid metabolism of zebrafish (*Danio rerio*).

In order to achieve this, a wide network of genes involved in lipid metabolism were examined, and the effect of *L. rhamnosus* on intestinal epithelial structures, total body cholesterol, TAG and non-polar fatty acids and zebrafish larval growth was investigated.

## Results

### Probiotic modulation of the digestive tract microbiome

**Figure 1:**
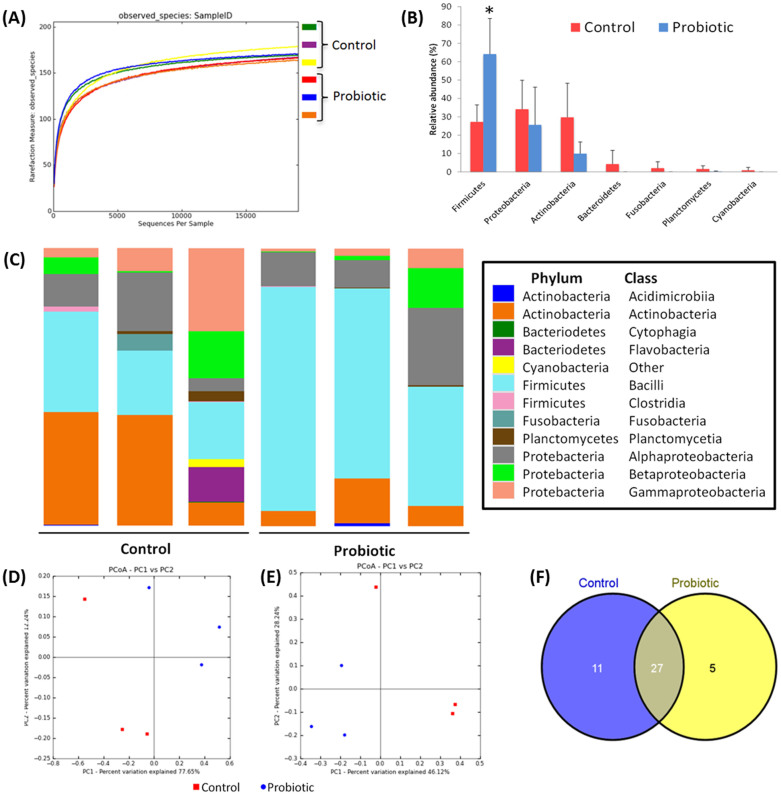
Digestive tract bacterial community analysis of 6 dpf zebrafish larvae. (A) Alpha rarefaction plot of observed species. (B) Relative abundance of reads assigned to phyla. (C) Stacked bar chart representing the relative abundance of bacterial phylum and classes. (D) and (E) Principal coordinates analysis (PCoA) plots using Bray-Curtis metric and weighted Unicfrac distances, respectively. (F) Unique and shared OTUs (at the level of genera) present in the treatment groups.

High-throughput sequence analysis of bacterial 16S rRNA V3 region was conducted on samples at 6 dpf. The analysis revealed a highly diverse microbiota with a total of 200 thousand unique reads (with an average length of 166.0 ± 6.5 bp), representing 218 OTUs, from over 1.2 million raw reads. Using default parameters, a *de novo* UCHIME algorithm^20^ was used to identify potential chimeric sequences, which accounted for 5.1% of the total sequences. The alpha rarefaction plot of observed species reached a saturation phase at approximately 169 OTUs indicating that adequate sequence coverage was obtained to reliably describe the full diversity present in the samples (Figure 1A). This was verified by the Good's coverage estimation values of >99.9% (Table 1). The bacterial branch distance, which measures the complete phylogenetic diversity represented within a community^21^, demonstrated a significantly lower microbial diversity in the probiotic group (2.34 ± 0.14) compared to control group (2.65 ± 0.16) (*P* < 0.05) (Table 1). In order to evaluate relationships among samples based on differences in phylogenetic diversity, two-dimensional principal coordinate analysis (PCoA) plots were calculated from weighted UniFrac distances for the evaluation of the community composition^22^ (Figure 1D) and Bray-Curtis metrics were used for the estimation of the dissimilarity among samples^23^ (Figure 1E). Both PCoA plots show clustering of the replicates from the probiotic treatment away from the control samples, suggesting that the probiotic modified the bacterial communities in a characteristic direction.

In general, the bacterial communities in both treatments were dominated by three phyla: Firmicutes, Proteobacteria and Actinobacteria. The distinguishable separation of bacterial communities found in PCoA was accompanied with differences in the composition of the gut microbiota. A small proportion of the reads were identified as belonging to the Bacteriodetes (4.3%), Fusobacteria (2.0%), and Planctomycetes (1.6%) phyla in the control, which were considerably less abundant in the probiotic group (where Bacteriodetes and Fusobacteria were not detected and Planctomycetes = 0.3% of the reads) (Figure 1B), and higher relative abundance of Firmicutes was observed in the probiotic group (*P* < 0.05, Figure 1B). Most OTUs were resolved to class-level, others by order and some to genus level (Figure 1B–C). Firmicutes were dominated by the class Bacilli, with this class more abundant in the treated group than the control (*P* < 0.05). *Lactobacillus* spp. were significantly more abundant in the probiotic treated larvae (accounting for 33.0% of the total reads) than the control larvae (accounting for 8.1% of the reads) (*P* < 0.05), BLAST results identified the dominant lactobacilli as *L. rhamnosus* (NR-102778) in the probiotic treated samples, accounting for 100% of the lactobacilli reads, whereas lactobacilli reads from the control comprised *Lactobacillus reuteri* and *L. rhamnosus*. The abundance of reads assigned to the *Streptococcus* genera did not differ significantly between the groups, but BLAST revealed that the species composition of this genera differed between the treatment groups: *Streptococcus thermophilus* (NR- 074827) dominated the probiotic samples and the control samples were dominated by an uncultured *Streptococcus* sp. most closely related to *Streptococcus sanguinis* (94% alignment similarity). A core microbiome of OTUs, detected in both treatment groups, was identified and comprised 27 OTUs (Figure 1F); these were the *Streptococcus, Pseudomonas, Lactobacillus, Acidovorax, Actinomyces, Enhydrobacter, Ochrobactrum, Phenylobacterium, Acinetobacter, Escherichia, Aminobacter, Corynebacterium* and *Agrobacterium* genera and 14 additional OTUs which could not be accurately identified at the genus level. Five OTUs were unique to the fish treated with the probiotic (*Janthinobacterium, Gluconacetobacter, Faecalibacterium* and two unidentified OTUs) and the 11 OTUs were uniquely detected in the control fish (*Chryseobacterium, Sneathia, Mycobacterium, Kocuria, Hymenobacter, Finegoldia* and five unidentified OTUs) (Figure 1F).

### *L.* *rhamnosus* affects the expression of genes involved in lipid metabolism

The high-throughput sequencing results evidenced a change in gut microbiota community exposed to *L. rhmanosus*. In order to gain further insight, we next wanted to evaluate effect of microbiota modulation on lipid metabolism, and thus the expression of genes related with lipid pathways were assessed.

**Figure 2:**
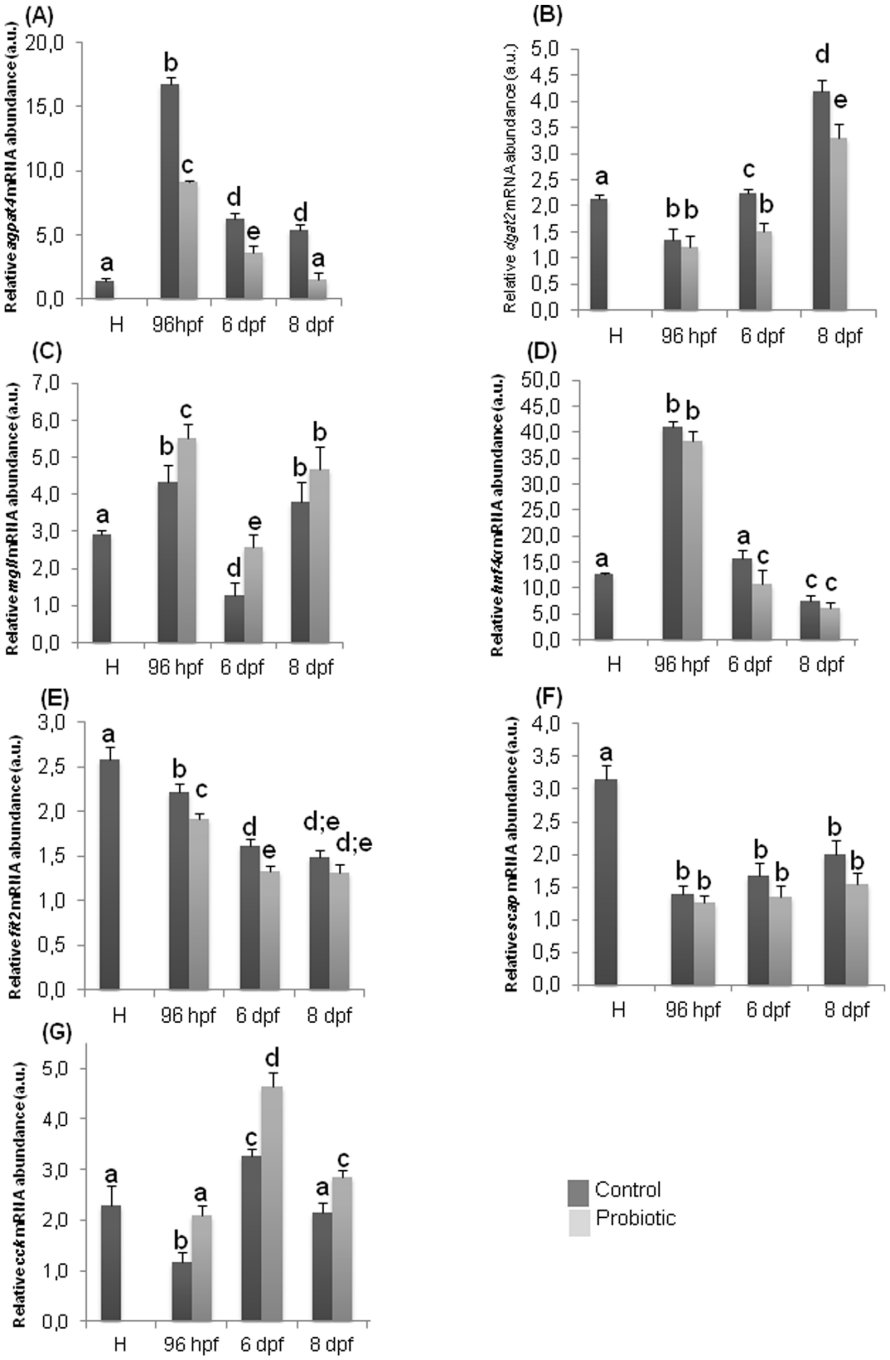
*L. rhamnosus* treatment modulates expression of genes involved in lipid metabolism. qRT-PCR analysis. Relative *agpat* (A), *dgat2* (B), *mgll* (C), *hnf4α* (D), *fit2* (E), *scap* (F), *cck* (G) gene expression normalized against *β-act* and *arp*, in pools of 15 zebrafish larvae from control and probiotic groups collected at hatching (H), 96 hpf, 6 dpf and 8 dpf. Assays were performed in triplicate. Values with different superscript letters are significantly different (*P* < 0.05).

At hatching, when probiotic administration was not yet supplied, the gene expression levels of *agpat4,* a gene encoding for an enzyme involved in the conversion of lysophosphatidate to phosphatidate in the glycerol phosphate pathway^24^, was almost undetectable (Figure 2A). During larval development of the control fish this gene reached the highest level of expression at 96 hpf and significantly decreased at 6 and 8 dpf (the last stage analyzed in the present study). A similar trend was recorded in *L. rhamnosus* treated fish with the highest levels of *agpat4* gene expression detected at 96 hpf and significantly lower levels at 6 and 8 dpf. Comparison between the two experimental groups revealed a significant down-regulation of *agpat4* in the probiotic treated larvae with respect to the control group, in all the developmental stages analyzed (96 hpf, 6 dpf and 8 dpf).

The gene expression analysis of *dgat2*, a gene encoding for a protein that catalyzes a reaction which produces TAG from DAG and fatty acyl-CoA^8^, was clearly detectable at hatching (Figure 2B); at 96 hpf a significant decrease occurred and a successive significant increase at 6 dpf and 8 dpf was observed. In treated larvae, the lowest *dgat2* level was detected at 96 hpf, gradually increasing at 6 dpf and reaching the highest level at 8 dpf. The comparison between control and treated groups evidenced similar levels at 96 hpf, while significantly lower levels were observed in the probiotic treated group at 6 and 8 dpf.

The analysis of *mgll* expression levels, which encodes for a serine hydrolase that preferentially hydrolyses monoacylglycerols [*e.g*. 2-arachidonoylglycerol; (2-AG)] to free fatty acid and glycerol^14^, evidenced its presence at hatching (Figure 2C). In the control, the expression was significantly higher at 96 hpf, significantly decreased at 6 dpf and a successively significant increase occurred at 8 dpf. The same trend was observed in treated larvae at all the developmental stages analyzed. The comparison between the two experimental groups revealed that probiotic treatment induced significantly higher expression at 96 hpf and 6 dpf while no difference was apparent at 8 dpf.

The analysis of *hnf4α*, a gene encoding for a protein implicated in the metabolism of cholesterol, fatty acids and amino acids^25^, evidenced its presence at hatching, and in both control and probiotic treated groups peaked at 96 hpf, and subsequently significantly decreased at 6 dpf reaching the lowest level at 8 dpf (Figure 2D). Compared to the control, *hnf4α* gene expression was down regulated by the probiotic at 6 dpf.

The *fit2* gene, encoding for a protein involved in fat storage^19^, transcript analysis in the control group showed the highest level at hatching; similar levels were maintained at 96 hpf followed by a significant decrease at 6 and 8 dpf (Figure 2E). In treated larvae, higher levels were observed at 96 hpf and 8 dpf while at 6 dpf the lowest levels were detected. Comparing the levels of *fit2* gene between the treatments, lower levels were observed at 96 hpf and 6 dpf in the probiotic treatment.

The analysis of the expression of *scap* (Figure 2F), encoding for Cleavage Activation Protein which activates SREBPs which regulate the transcription of genes involved in uptake and synthesis of *de novo* cholesterol and fatty acids^26^, evidenced highest levels at hatching. In both experimental groups a significant decrease of gene expression was detected at all stages analysed compared to the levels at hatching. No significant differences were observed between the control and probiotic groups at any of the developmental stages analyzed.

Finally the analysis of *cck*, which encodes for a peptide hormone responsible for gallbladder contraction and pancreatic enzyme secretion^27^, was clearly detectable at hatching (Figure 2G); at 96 hpf a significant decrease was detected and a successive significant increase at 6 dpf was observed in the control group compared to the time of hatching. Finally, at 8 dpf, the gene expression level reached the same level of that at the time of hatching. The same trend was observed in treated group. The comparison between control and treated groups evidenced significantly higher levels in the probiotic treated group in all the developmental stages analyzed (96 hpf, 6 dpf and 8 dpf).

### Modulation of microbiota decreased the total body cholesterol and triglyceride levels

Triglycerides and cholesterol derived from the diet are used from both fish and mammals as the main source of energy which can be released rapidly on demand^6^. TAGs represent the major source of lipid storage and cholesterol and are an important component of cell membranes; they are also the precursor molecules for the synthesis of steroid hormones, bile salts and vitamin D^28^,^29^. However, it has been reported that excess TAG storage as well as high serum cholesterol levels cause several metabolic disorders including Type 2 diabetes, hypertension and atherosclerosis^30^. To gain further insight into the probiotic effects on lipid metabolism, the zebrafish total cholesterol and TAG content were evaluated. The High Performance Liquid Chromatography (HPLC) analyses performed on total body samples evidenced that zebrafish treated with *L. rhamnosus* had significantly lower levels of total cholesterol (0.083 ± 0.006 μg/mg) and TAG (0.040 ± 0.003 μg/mg) compared to the total cholesterol (0.131 ± 0.014 μg/mg) and TAG (0.088 ± 0.010 μg/mg) levels in the control group (*P* < 0.05) (Table 2).

### Ultrastructural analysis by Transmission Electron Microscopy (TEM) revealed longer enterocytes and microvilli heights in the intestine of probiotic treated zebrafish

**Figure 3:**
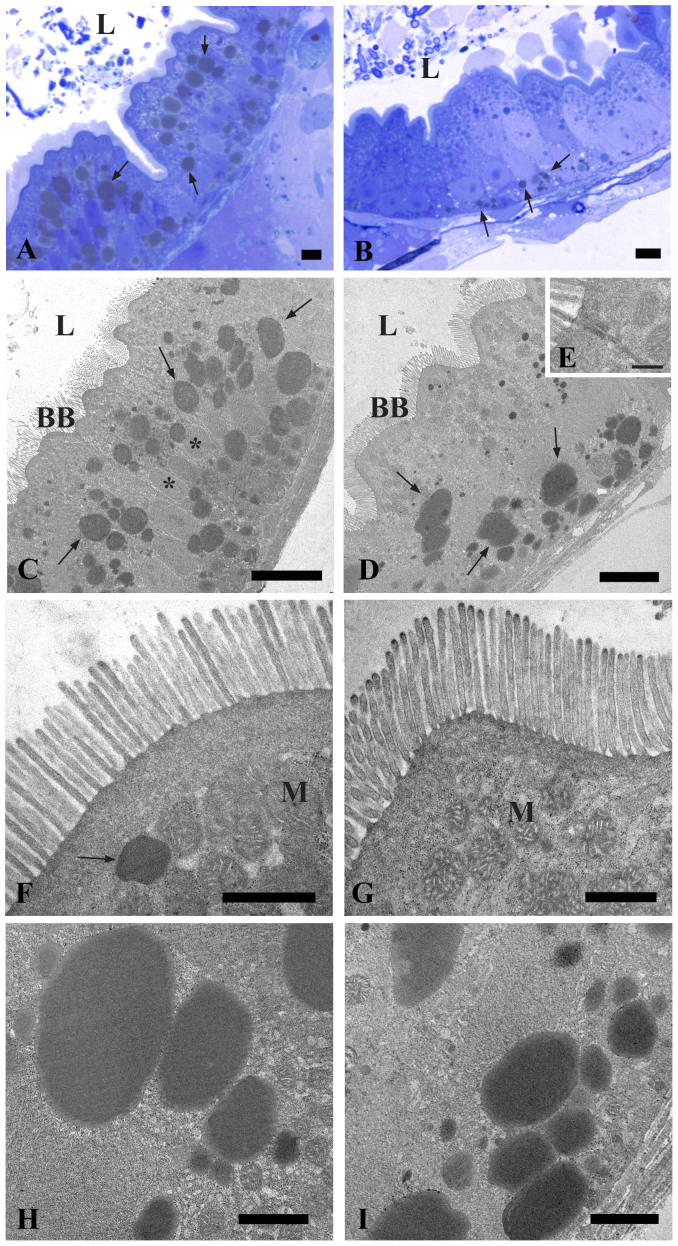
Transmission Electron Microscopy (TEM) shows the ultrastructure of the intestine in control and probiotic treated zebrafish. Thin section (80 nm) of 6 dpf zebrafish showing the epithelial layer arranged into broad, irregular folds (A). Intact epithelial barrier, lack of cell debris in the lumen and no signs of damage in larvae exposed to probiotic *L.* *rhamnosus* (B). Electron micrographs showing uniform, columnar, polarized epithelia, with an apical brush border in control (C) and probiotic treated groups (D). Higher magnification of junctional complexes (E). Microvilli on the apical surface and copious spherical mitochondria in the enterocyte cytoplasm of control (F) and treated larvae (G). Higher magnification of the lipid droplets in the enterocytes of control larvae (H) and in the probiotic treated intestine (I). BB: brush border; L: lumen; M: mitochondria; *: nucleus; arrows: lipid droplets. Scale bar: 5 μm in (A, B); 10 μm in (C, D); 500 nm in (E), 1 μm in (F, G); 2 μm in (H, I). (See also Figure S3).

At 6 dpf the alimentary canal of zebrafish comprises the mouth, pharynx, esophagus, intestinal bulb, mid-intestine, posterior intestine and anal opening. The yolk is completely resorbed^31^. In the intestinal bulb, the rostral region of the gut, the lumen was greatly expanded and the epithelial layer was arranged into broad, irregular folds. The intestine of control group had an undamaged epithelial barrier and no signs of degradation (Figure 3A). The intestines exposed to the probiotic *L.* *rhamnosus* had an intact epithelial barrier, lack of cell debris in the lumen and no signs of damage (Figure 3B). At this stage the intestine consisted mainly of uniform, columnar, polarized epithelia, with an apical brush border, lateral cell border, and basal basement membrane. Both in control and treated groups (Figure 3C–D), columnar-shaped enterocytes were joined apically through a complex set of junctional complexes (tight junctions, adherens junctions and desmosomes) that restrict the movement of membrane components between apical and basolateral cellular domains, and serve as a barrier to the paracellular space through which luminal contents may otherwise enter the organism (Figure 3E). In the probiotic treated larvae the enterocytes lengths were significantly higher (42.24 ± 1.78 μm) compared to the control (34.64 ± 2.06 μm) and significantly longer microvilli on the apical surface were observed in *L.* *rhamnosus* exposed larvae (1.21 ± 0.09 μm) with respect to the control (1.06 ± 0.08 μm) (*P* < 0.05) (Figure 3F–G). The enterocyte‘s apical cytoplasm contained copious spherical mitochondria and endoplasmic reticulum. In addition, lipid accumulations were present in the apical and basal cytoplasm of both control and treated groups (Figure 3 C–D; H–I). These accumulations were determined to be lipid droplets by their uniform interior, association with endoplasmic reticulum and mitochondria, and presence of a bounding monolayer membrane. In probiotic treated larvae the diameter of the lipid droplets (2.37 ± 0.37 μm) was significantly smaller compared to the control (4.10 ± 0.40 μm) (*P* < 0.05). *Supplemental material*: see also supplemental Figure S3 (A) for enterocyte lengths (related to Figure 3 C–D), Figure S3 (B) for lipid droplet diameter (related to Figure 3 C–D; H–I), and Figure S3 (C) for microvilli lengths (related to Figure 3 F–G).

### *In vivo* non-polar fatty acids localization with BODIPY 505/515 staining revealed accumulations in the gallbladder and intestine of probiotic treated fish

**Figure 4:**
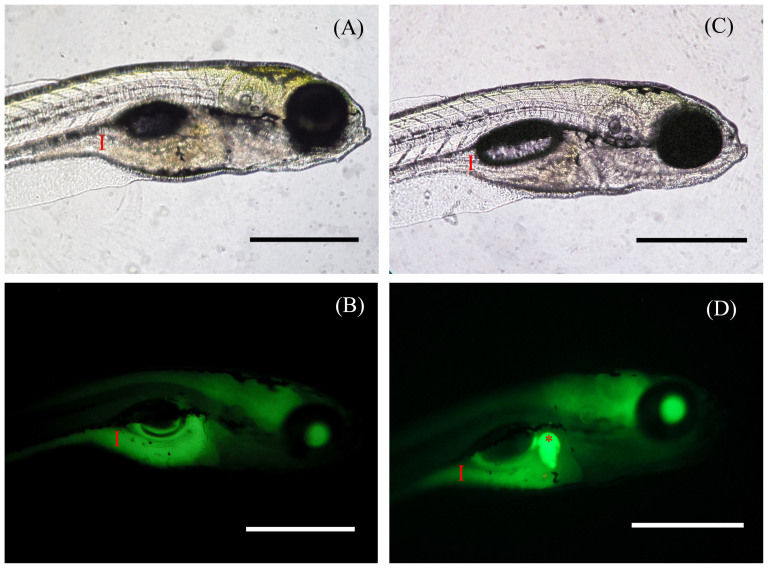
BODIPY 505/515 staining evidenced an accumulation of non-polar fatty acids in probiotic treated gallbladder. Representative fluorescent images of live 6 dpf zebrafish soaked in BODIPY 505/515 for 1 hour. Images revealed green fluorescence in the intestine in both control (A–B) and probiotic treated groups (C–D). Probiotic treated larvae exhibited higher fluorescent signal in the gallbladder (D). Red asterisk: gallbladder; red arrow: intestine. Scale bar: 500 μm. (See also Figure S4).

Teleosts and mammals have complex digestive physiologies but share a high degree of anatomical similarity^32^. Zebrafish, like humans, use TAG as a major source of lipid, and in order for the intestine to absorb TAG, processing by digestive enzymes and bile is required^33^. Bile and enzymes pass from the gallbladder and arrive to the intestinal lumen where TAG are emulsified and form micelles and finally, free fatty acids (FFAs) and monoacylglycerols are taken up by intestine^34^. It has been demonstrated that FFAs have an important role in gallbladder secretion^35^. We used BODIPY 505/515 (borondipyrromethene fluorescent moiety) staining as a non-invasive *in vivo* method in order to visualize the dynamics of non-polar fatty acids in living tissues by confocal microscopy. Imaging revealed similar green fluorescence intensity in the intestine of both control (320 ± 34 a.u.) (Figure 4 A–B) and probiotic treated zebrafish larvae (338 ± 52 a.u.) at 6 dpf (Figure 4 C–D) (*P* < 0.05). On the contrary, probiotic treated larvae exhibited significantly higher fluorescent signal in the gallbladder (535 ± 66 a.u.) with respect to the control (340 ± 53 a.u.) (*P* < 0.05) highlighting an accumulation of non polar-fatty acids.

*Supplemental figure*: Figure S4 (E) shows graph with statistical difference of intestinal fluorescence of both control and treated larvae (Figure 4 A–B–C–D).

### Treatment with *L. rhamnosus* improves the growth of zebrafish larvae

**Figure 5:**
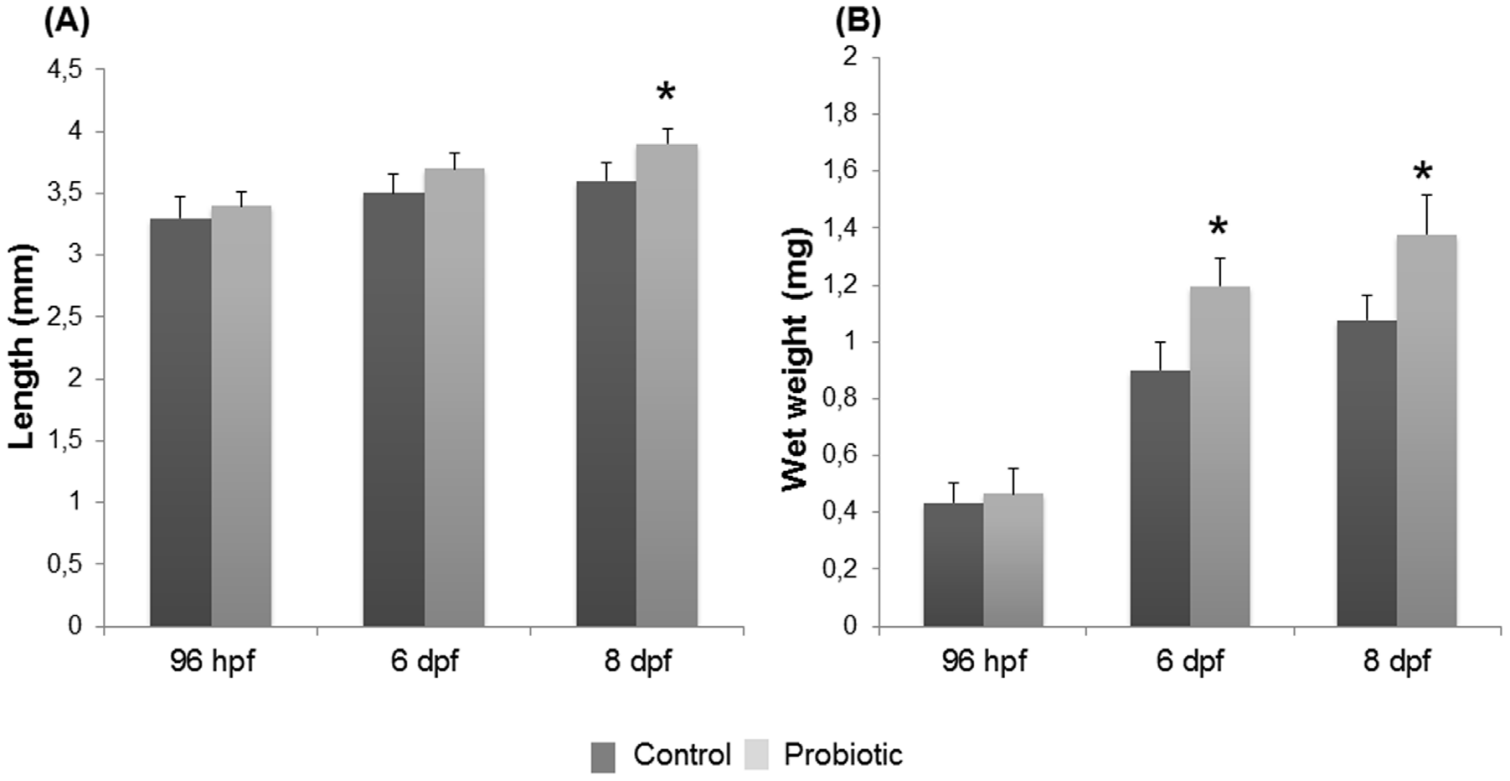
*L. rhamnosus* administration increased larvae total length and body weight. Total length (A) and wet weight (B) of zebrafish larvae from control and probiotic groups collected at 96 hpf, 6 dpf and 8 dpf. Data are the mean ± s.d. Asterisk indicates significant differences (*P* < 0.05).

Since we reported compelling evidence of gut community modulation and in turn lipid metabolism modulation after administration of *L. rhamnosus*, by measuring total larval length and wet weight, we sought to determine if these changes impacted the growth of zebrafish larvae. At 96 hpf no significance differences were observed in terms of length and weight in both control (length 3.3 ± 0.17 mm; weight 0.43 ± 0.07 mg) and treated (length 3.4 ± 0.11 mm, weight 0.4 ± 0.08 mg) larvae (*P* > 0.05). At 6 dpf no significant difference of larval length was observed between control larvae (3.5 ± 0.16 mm) and larvae exposed to *L. rhamnosus* (3.6 ± 0.18 mm) (*P* > 0.05). Despite this, a significant increase of wet weight was observed at 6 dpf in the probiotic supplemented group (1.19 ± 0.09 mg) compared to the control group (0.9 ± 0.1 mg). At 8 dpf a significant increase of total length (3.9 ± 0.12 mm) concomitant with a significant increase of wet weight (1.37 ± 0.13 mg) was apparent in zebrafish larvae treated with the probiotic compared to the length (3.6 ± 0.21 mm) and wet weight (1.07 ± 0.08 mg) detected in control group (*P* < 0.05) (Figure 5A–B). In all stages examined the viable larvae observed in both control and treated groups were 95–98%.

## Discussion

In the current study, the administration of *L. rhamnosus* induced changes to the microbial composition of the zebrafish digestive tract. The probiotic treatment reduced the presence of some genera which contain potential pathogens (e.g. *Mycobacterium*) and enhanced the abundance of reads assigned to the class Bacilli. Reads from the *Lactobacillus* genera were significantly elevated by the probiotic larvae and in addition the probiotic enhanced the presence of another lactic acid bacteria, *Streptococcus thermophilus*. This finding is in agreement with the previous study on 6-month old zebrafish fed dietary *L. rhamnosus* which demonstrated that the probiotic populated the intestinal tract and elevated the abundance *Streptococcus thermophilus*^36^. *Streptococcus thermophilus* and lactobacilli (*e.g*. *Lactobacillus delbrueckii* subsp. *bulgaricus*) are known symbionts as observed from their interactions as starter cultures in dairy products; *Streptococcus thermophilus* is suggested to provide folic acid and formic acid to the lactobacilli which in turn provides a source of amino acids^37^. The present study, and that of Gioacchini *et al*.^36^, would suggest that a similar symbiotic relationship may occur between *L. rhamnosus* and *S. thermophilus* in the digestive tract of zebrafish. Indeed, some strains of lactobacilli and streptococci have been reported to have symbiotic effects in mammals, subsequently reducing hepatic cholesterol levels in rats fed a fat- and cholesterol-enriched diet^38^.

The *in vivo* mechanisms of the gut microbiota which influence host dietary lipid metabolism are still unknown, however Semova *et al*.^1^ identified that colonization of the zebrafish gut with Firmicutes promotes epithelial absorption and is involved in host energy balance of zebrafish larvae. Further studies are required to enhance our understanding of the roles that gut microbes play in lipid metabolism and specific studies should elucidate the extent to which the observed effects in the present study are *L. rhamnosus*-specific; for example, it is not apparent from the present study if the host effects could be driven by overall changes, elevations in microbial load or whether other probiotic species are capable of driving these effects.

Microvilli and enterocyte height are directly correlated with the gut function and health of the host; an increase of microvilli and enterocytes heights provide increased absorptive surface area. Our TEM analysis highlighted increased microvilli and enterocyte heights, suggesting that *L. rhamnosus* is able to expand intestine structures. These findings are in agreement with previous probiotic studies^39^,^40^ and are likely to contribute towards the improved growth performance of the zebrafish larvae in the present investigation. Additionally, it has been observed that gut microbiota can ameliorate the absorption of nutrients and minerals, in particular calcium^41^. In the intestine, microbial production of SCFAs, which may have been elevated by the probiotics, can decrease the pH which indirectly increases the solubility of minerals and subsequently their absorption^42^. Furthermore, the symbiotic relationship between *L. rhamnosus* and *S. thermophilus* and their ability to generate metabolites, such as SCFAs, which can allow the host, especially the enterocytes, to use energy from the diet in a more efficent way. Is well known that enterocytes obtain a large proportion of their energy from the oxidation of luminally derived SCFAs and absorbed SCFAs not utilised by enterocytes may also provide additional energy at the systemic level for other cell types^43^. These events could positively affect the host, leading to an increase in host growth^44^.

Further, possible modulation of the expression of genes involved in lipid metabolism may also have contributed towards improved growth.

*L. rhamnosus* administration and the relative modulation of the gut microbiota was able to induce transcriptional changes to a suite of genes involved in the synthesis, transport, storage and homeostasis of lipids, in particular, cholesterol and TAG. TAG biosynthesis is catalysed by AGPAT4 and DGAT2 enzymes and recent studies in mice and rats showed that their inhibition decreases TAG synthesis^45^,^46^,^47^. In this study, *agpat4* and *dgat2* transcription were both decreased by *L. rhamnosus*. Furthermore these transcriptional changes induced by *L. rhamnosus* administration reflected a significant reduction in TAG content observed in treated larvae. TAG, together with sterol esters and phospholipids, form LD and recent studies on mouse liver and muscle evidenced that knockdown of *fit2* significantly decreases LD accumulation^48^. Recently, it was demonstrated that *fit2* expression is ubiquitous in all zebrafish tissues^49^. In the present study, the lower level of *fit2* mRNA found in probiotic treated larvae was concomitant with the reduction in size of LD in the intestine of treated larvae, evidenced by TEM analyses. This result is consistent with a study showing that *fit2* knockdown in zebrafish resulted in a decrease of LD in the liver and intestine^49^.

Recently, Guo and co-workers^50^ demonstrated that stimulation of lipolysis may break down large LDs into smaller LDs, in order to increase the surface area available for lipases, which are able to hydrolyze TAG. We observed a reduction in triglyceride content and a decrease of LDs accumulation, suggesting that *L. rhamnosus* affects the expression of TAG metabolism related genes (*agpat4*, *dgat2* and *fit2*) and may stimulate lipolysis. Our findings on LDs accumulation are also consistent with a previous study in *Solea senegalensis* which showed the ability of a probiotic from the *Alteromonadaceae* family to inhibit intestinal LDs accumulation^51^. Interestingly, BODIPY 505/515 staining evidenced an intense fluorescent signal in the gallbladder of probiotic treated larvae, indicating that this non-polar lipid can be absorbed by the intestine, successively taken up by the liver and secreted into the gallbladder, through hepatobiliary transport.

The enhancement of fluorescence in the gallbladder prompted us to speculate an accumulation of non-polar fatty acids and/or other hydrophobic region that constitute most of the lipids. The bile in the gallbladder is composed by several molecules and among these, bile acids and bile alcohol, composed by both hydrophobic and hydrophilic regions, are the most abundant molecules in the bile of vertebrates^52^. Zebrafish bile contains bile alcohol^52^. The synthesis of bile alcohol is not thought to be influenced by diet, rather mostly regulated by the gut microbiota, which possess unique enzymes involved in deconjugation, oxidation and esterification of bile alcohol^53^; thus, this result could indicate an increase of non-polar lipid bile fraction production as a result of the probiotic activity, as previously demonstrated by Pavlović *et al*.^54^

It has been evidenced that free fatty acids stimulate CCK, which induces the contraction of the gallbladder to release bile into the intestine and solubilize lipids into micelles that are hydrolized by pancreatic lipase^35^. Our results demonstrate an up-regulation of *cck* gene expression levels concomitant with a higher content of non-polar fatty acids in the gallbladder after the probiotic supplementation. This could indicate increased bile production and hepatobiliary function, reduced bile secretion into the intestine, altered intestinal motility rates, improved the enterohepatic cycling of bile acids and/or bile transportation^55^,^56^.

Recently, MGLL has been described as one of the lipases involved in the final step of TAG hydrolysis^57^. Our results showed an up-regulation of *mgll* gene expression level that reflected the lower TAG content observed, consistent with the presence of non-polar fatty acids (as a probable result of the breakdown of triglycerides) in the gallbladder after probiotic supplementation, which indicates the role of the digestive tract microbiota in the modulation of non-polar fatty acids metabolism.

In addition, recent studies showed that *hnf4α* knock-down reduces cholesterol content^58^,^59^. Our data indicate that *L. rhamnosus* treatment decreases the expression of the *hnf4α* gene, and therefore these results suggest that the modulation of gut microbiota, by inducing transcriptional changes, modulates cholesterol metabolism. The down-regulation of *hnf4α* after probiotic supplementation reflected a lower cholesterol content in the larvae. These findings are in agreement with a study which demonstrated cholesterol-lowering action by probiotics in mammals^60^. Cholesterol homeostasis is controlled by *scap* which plays an essential role in maintaining and controlling *de novo* synthesis of cholesterol, and therefore, is responsible for endogenous biosynthesis^61^. The maintenance of *scap* levels in the current study, concomitant with the decrease of cholesterol in treated larvae, suggests that the cholesterol reduction is not related with lower *de novo* synthesis but with its consumption.

The modulation of cholesterol metabolism evidenced in our study, could be due to the production of SCFAs as final products of bacterial activity in the digestive tract: SCFAs can decrease lipid levels in the blood by blocking the synthesis of hepatic cholesterol and transferring plasma cholesterol to the liver^4^,^62^.

This study clearly evidenced a novel role of *L. rhamnosus* on the modulation of the microbiota community in the zebrafish digestive tract. The digestive tract microbiome changes in response to the probiotic supplementation modulated the expression of a network of genes involved in the physiological control of lipogenesis, a concomitant reduction of total body cholesterol and triglycerides, an increase of non-polar fatty acids, an improvement of the intestinal epithelium structures (*i.e*. microvilli and enterocytes) and a reduction in enterocyte LDs, together with an increase in growth of probiotic treated zebrafish larvae.

The findings we report provide a novel gene network through which *L. rhamnosus* acts and, by inducing transcriptional changes, modulates lipid metabolism. Probiotic *L. rhamnosus* also i) modulates gut microbial composition, ii) increases FAs concentration, and iii) expands the absorptive surface of the intestinal epithelium by increasing microvilli and enterocytes heights. Since the zebrafish has become an established vertebrate model for a wide range of human diseases with particular strengths for biomedical research, the results discussed here suggest a possible use of *L. rhamnosus* for improving lipid metabolic disorder.

## Methods

### Animals and probiotic administration

Adult female and male zebrafish (*D. rerio*) were purchased from Acquario di Bologna (Italy) and acclimatized to the laboratory conditions (27.0 ± 0.5°C under a 12:12 h light:dark photoperiod). Pairs were spawned individually and larvae were raised under a 12:12 h light:dark cycle at 27°C. Embryos were collected and after hatching were divided into a control group and a probiotic-treated group. Larvae were fed a commercial diet (JBL flakes, Germany) consisting of 43.00% crude protein, 8.30% crude fat, 8.10% ash, 1.9% fibre and 8.00% moisture content. The probiotic treatment consisted of the administration of *L. rhamnosus* IMC 501® (C025396A; Synbiotec, Camerino, Italy) via the rearing water at a concentration of 10^6^ colony-forming units (CFU) according to previous studies^36^,^63^,^64^,^65^.

The experiment was set up in triplicates, with three control tanks and three probiotic tanks and from each tank a pool of larvae was collected. The experiment was repeated three times.

Both control and probiotic groups were fed twice a day. At hatching, 96 hpf, 6 dpf and 8 dpf, larvae were euthanised using MS222 (100 mg L^−1^) (Sigma-Aldrich) and stored at −80°C for real time PCR analyses. At 96 hpf, 6 and 8 dpf larvae were sampled for morphometric analyses. Since at 6 dpf, molecular analysis revealed significant changes of expression of all genes analysed, the high-throughput sequencing, HPLC, TEM and BODIPY staining were performed at this stage of development. Larvae from all stages, both control and the treated group were sampled 18 hrs post meal, at 8 am. All procedures involving animals were conducted in accordance with the Italian law on animal experimentation and were approved by the Ethics Committee of Università Politecnica delle Marche (Prot #63/INT/CESA12-16).

### RNA extraction and cDNA synthesis

Total RNA was extracted from 15 whole larvae per tank using an RNAeasy® minikit (Qiagen, UK) according to Maradonna *et al.*^66^.

### Real time PCR

PCRs were performed with the SYBR green method in an iQ5 iCycler thermal cycler (Bio-Rad laboratories). Triplicate PCRs were carried out for each sample analyzed. The reactions were set following Maradonna *et al.*^66^. The data obtained were analyzed using the iQ5 optical system software version 2.0 (Bio-Rad laboratories). Modification of gene expression is reported with respect to the control sample. The primer sequences for *actβ, rplp*, *mgll, hnf4α*, *fit2, scap* and *cck* were designed using Primer3 (210 v. 0.4.0), primers for *agpat4* and *dgat2* were taken from Her *et al.*^67^.

The primer sequences are: *agpat4* forward TGCTGAAAACTCAGTTGCTG, *agpat4* reverse AGTAACCCAGTCTGCAGTTG, *dgat2* forward TTCCGGTGTCAAAAAGGGCT, *dgat2* reverse CAGCAGCAAAGAGCAAGCAA, *mgll* forward CGAGAGGCCGCAGGATTTTA*, mgll* reverse TGAGTTTAGGAGCCAAGCG, *hnf4α* forward ACGGTTCGGCGAGCTGCTTC, reverse *hnf4α* TCCTGGACCAGATGGGGGTGT, *fit2* forward CTGGTCTCCCTCCACAGCCGA, *fit2* reverse ACACCAGCTGCCCTCCGCTT, *scap* forward GCTGTTACCCTCTGCTGAAG, *scap* reverse TGAAACCGCTGCCTTGAC, *cck* forward CGCCTGCTGGACAAATCAAC, *cck* reverse TGCGGTATGAGCCTTTGGTT, *rplp* forward CTGAACATCTCGCCCTTCTC-3′, *rplp* reverse TAGCCGATCTGCAGACACAC, *β-act* forward GGTACCCATCTCCTGCTCCAA, *β-act* reverse GAGCGTGGCTACTCCTTCACC.

## Digestive tract microbiome analysis

### DNA extraction and PCR

DNA was extracted from 100 mg of the sterilized organisms, after a 30 min lysozyme incubation (50 mg mL^−1^) at 37°C, using a QIAamp® Stool Mini Kit (Qiagen, Crawley, UK) according to the manufacturer's instructions, with some modifications in the cleanup and precipitation steps according to^68^. Briefly, after the removal of proteins, 460 μL of extract product was added to an equal volume of ice-cold Tris-buffered phenol solution, left to stand on ice for 10 min, then washed twice with an equal volume of chloroform, and centrifuged for 5 min at 3,000 g. The upper aqueous layer was isolated and the DNA precipitated overnight using an equal volume of ice-cold isopropanol and 10% sodium acetate, before centrifugation at 14,000 g for 30 min at 4°C. The DNA pellet was washed twice using 300 μL of 70% molecular biology-grade ethanol, before air drying for 5 min, and resuspension overnight at 4°C in 30 μL sterile water. The DNA concentration was determined at 260 nm using a NanoDropTM 1000 spectrophotometer (Thermo Scientific Ltd, DE, USA). All reagents used were molecular grade, purchased from Sigma-Aldrich (UK).

PCR amplification of the 16S rRNA V3 region was conducted with a nested PCR strategy^69^, the external PCR was carried out using primers Eub8F (5′-AGAGTTTGATCMTGGCTCAG-′3)^70^ and 984yR (5′-GTAAGGTTCYTCGCGT-3)^71^; the internal PCR was conducted using the primers P1F (5′-CCTACGGGAGGCAGCAG-3′ + GC clamp) and P2R (5′-ATTACCGCGGCTGCTGG-3′)^72^. Two μL of DNA template was used in both reactions. The PCRs were performed in a Techne TC-312 cycler (Thermal Cyclers, Staffordshire, UK) for both reactions with 25 cycles of 95°C for 30 s, 50/53°C for 30 s, 72°C for 1 min and a final extension 72°C for 5 min. The annealing temperatures were 50°C and 53°C for the external and internal PCRs, respectively.

### High-throughput sequence analysis

PCR products were purified using a QIAquick PCR Purification Kit and quantified using a Qubit® 2.0 Fluorometer (Invitrogen). Prior to sequencing the amplicons were assessed for fragment concentration using an Ion Library Quantitation Kit (LifeTechnologies™, USA), and concentrations were then adjusted to 26 pM. Amplicons were attached to Ion Sphere Particles (ISPs) using an Ion PGM™ Template OT2 200 kit (LifeTechnologies™, USA) according to the manufacturer's instructions. Multiplexed sequencing was conducted using a 316™ chip (LifeTechnologies™) on an Ion Torrent Personal Genome Machine (LifeTechnologies™). Sequences were binned by sample and filtered within the PGM software to remove low quality reads. Data were then exported as FastQ files.

Taxonomic analyses of sequence reads were performed after the removal of low quality scores (Q score <20) with FASTX-Toolkit (Hannon Lab, USA). Sequences were concatenated and sorted by sequence similarity into a single fasta file. Sequences were denoised and analyzed with QIIME^73^. Briefly, OTU mapping was performed using the USEARH quality filter pipeline^20^, to remove putatively erroneous reads (chimeras), then OTU picking was achieved with a minimum pairwise identity of 97%. The most abundant sequence in each OTU were selected to assign a taxonomic classification based on the Greengenes database^74^ using the RDP classifier^75^, clustering the sequences at 97% similarity with a 0.80 confidence threshold. PyNast was used to create a multiple alignment of the representative sequences for each OTU^76^ with minimum sequence length threshold of 120 bp and 97% identification. Sequences were filtered to remove outliers and filter positions with gaps (0.95) and singletons. Highest homologous species were identified at 97% and 120 bp using the resulting consensus sequences in CLC Bio Genomics Workbench v6.0.4 (QIAGEN, CLC Inc, Aarhus, Denmark) against the 16S microbial BLAST-NCBI database from 04/22/2013, after a *de novo* assembling with default parameters except that 80 bp was used as the minimum contig length.

Alpha diversity metrics were calculated on rarefied OTU tables with QIIME to asses sampling depth coverage using observed species, phylogenetic diversity (PD), Chao1, Shannon's diversity index and Good's coverage. QIIME was also used to calculate Beta diversity metrics among samples using weighted Unifrac distances^22^ and Bray-Curtis similarity^23^. The distance matrixes were represented by two dimensional principal coordinates analysis (PCoA) plots.

### High-performance liquid chromatography (HPLC)

The total lipid contents of 300 mg of larvae at 6 dpf were extracted with the chloroform-methanol method (2:1 v/v)^77^. Cholesterol and TAG were quantified by HPLC-Light Scattering Detector (LSD) analyses using a LC-10AD VP gradient pump (Shimadzu, Kyoto, Japan), a VisionHT Silica 3 μm column (150 × 2.1 mm i.d., Grace Davison Discovery Science, Deerfield, IL, USA) and a LSD (Sedex 55, S.E.D.E.RE., France), operating at 40°C and nitrogen pressure of 240 kPa. The chromatograms were acquired and the data handled using the Class-VP software (Shimadzu). A binary gradient system composed of hexane [eluent A] and 2-propanol [eluent B] was used following the solvent elution profile: 0 min 100%A, 10 min 90%A, 15 min 80%A, 20–30 min 100%A. The flow rate was 400 μL min^−1^. Identification of cholesterol and TAG in the samples was carried out by comparison of their retention time with those of the respective commercial standards. Repeated injections of standard solutions were carried out to test the analytical precision. The relative standard deviations were less than 5% for all the analyses, both considering the intradie precision, calculated on six repeated injections, and the interdie precision, evaluated over six days. Calibration curves, drawn for each lipid standard by injecting different concentrations (5–40 μg mL^−1^), were used to quantify cholesterol, cholesteryl esters and TAG in the fish samples. All the HPLC analyses were carried out in triplicate. Cholesterol (≥99%) and glyceryl trioleate (≥99%) were purchased from Sigma-Aldrich (St. Louis, MO, USA). Hexane and 2-propanol were of high-performance liquid chromatography (HPLC) grade and were obtained from Merck (Darmstadt, Germany). The data obtained have been normalized to DNA content.

### Transmission Electron Microscopy (TEM)

Samples of 10 zebrafish larvae at 6 dpf were fixed with 1% potassium dichromate, 1% osmium tetroxide and 2% glutaraldehyde in cacodylate buffer (0.1 M, pH 7.2) for 5 hours at 4°C. Samples were washed four times with cacodylate buffer for 12 hours at 4°C, then dehydrated with a graded acetone series (from 50% to 100%) and embedded in epon-based resin. All reagents were purchased from AGAR Scientific (UK). The thin (1 μm) and ultrathin sections (60–80 nm) were obtained with a Reichert Ultracut ultramicrotome using a diamond knife and were collected on glass slides or copper grids. The thin sections were stained with 1% toluidine blue and images were captured using a computer-assisted image analysis system which includes a Zeiss microscope equipped with a colour video camera (Axio Cam MRC, Arese Milano Italy). The ultrathin sections were stained with 1% uranyl acetate and Reynolds lead citrate and then observed by TEM (JEOL 1200 EXII, JEOL Tokyo, Japan). Micrographs were acquired with an Olympus SIS VELETA CCD camera equipped with the iTEM software. Microvilli lengths, enterocyte lengths and lipid droplet diameters were determined from 10 fish per group; 6 images were taken for each intestine and 12 microvilli and enterocytes were measured per image. Enterocyte lengths were measured starting from their basal membrane connected with lamina propria up to the base of microvilli, while microvilli were measured starting from their base to the apical tip. All measurements were taken from micrographs using Software Image J^78^. In all cases, measurements were made using sections in which enterocytes were cut in their entire length, with the nuclei visible and aligned, to ensure that the section plane was appropriate.

### BODIPY 505/515 staining

Fifteen larvae at 6 dpf were placed into 12-well plastic dishes (3 larvae per well) and soaked in 100 μM BODIPY 505/515 (4,4-Difluoro-1,3,5,7-Tetramethyl-4-Bora-3a,4a-Diaza-*s*-Indacene) (Invitrogen) diluted in 2% DMSO, then incubated for 1 hour at 28°C in the dark. Larvae were anaesthetized using MS222 (100 mg L^−1^) (Sigma- Aldrich, Milan, Italy) and fluorescent images were obtained by a Bio-Rad MRC 1024 confocal microscope (Bio-Rad Microscience Ltd, Bio-Rad House, Hemel Hempstead, UK); equipped with a 10× objective. Bodipy 505/515 was excited at 505 nm (blue light) and emitted a spectrum of wavelength light which peaked at 515 nm. Regions of Interest (ROI) of 15 galbladders and 15 intestines per group were selected. Fluorescence intensity (arbitrary units a.u.) was determined using Image J software.

### Morphological analysis

At each time point larval length was measured using a Stemi 2000 micrometric Microscope (Zeiss Vision Italia, Castiglione Orona, Italy) and weight was determined using a Microbalance (OHAUS Explorer E11140, Pine Brook, NJ, USA).

### Statistical analysis

Results were expressed as the mean ± s.d. Statistical differences were determined using ANOVA, followed by Bonferroni's multiple comparison test. All statistical analyses were performed using Prism 6 (GraphPad Software, San Diego, CA, USA). A t-test was used to identify significant differences in relative abundance of bacterial taxa. P-values <0.05 were considered significant.

## Author Contributions

Conceived and designed the experiments: S.F. and O.C. Performed the experiments: S.F., F.M., A.R.T., G.G., A.R., S.P., L.C. and I.O. Analyzed the data: S.F., S.P., A.R., D.M. and O.C. Contributed reagents/materials/analysis tools: S.P., D.M., A.R. and O.C. Wrote the paper: S.F., S.P., D.M., A.R. and O.C.

## Supplementary Material

Supplementary Information supplementary information

## Figures and Tables

**Table 1:** Alpha diversity metrics of observed species, phylogenetic diversity (PD), Chao1, Shannon's diversity and Good's coverage of zebrafish larvae of 6 dpf (mean ± s.d.; n = 3)

	Observed species	Chao1	Shannon	Phylogenetic distance	Good‘s coverage
**Control**	195.1 ± 9.2	209.3 ± 12.2	5.57 ± 0.37	2.74 ± 0.14^b^	0.9992 ± 0.0002
**Probiotic**	194. 5 ± 1.5	206.5 ± 1.1	5.92 ± 0.14	2.45 ± 0.05^a^	0.9994 ± 0.0001

Values with different superscripts, within the same column, are significantly different at *P* < 0.05.

**Table 2:** *L. rhamnosus* administration affects whole larval cholesterol and triglycerides content

Parameter	Control	Probiotic	
	Value	Value	P-value
**Cholesterol (μg/μg DNA)**	0.131 ± 0.014	0.083 ± 0.006*	0.04
**TAG (μg/μg DNA)**	0.088 ± 0.010	0.040 ± 0.003*	0.02

Probiotics significantly decrease the content of cholesterol and triglyceride (TAG) in 6 dpf zebrafish larvae (pools 300 mg of larvae). Data are the mean ± s.d. Data have been normalized with DNA content. Values with asterisk are significantly different (*P* < 0.05).
