# Supplementary material for: Lactobacillus rhamnosus lowers zebrafish lipid content by changing gut microbiota and host transcription of genes involved in lipid metabolism
Source: Sci Rep. 2015 Mar 30;5:9336. doi: 10.1038/srep09336 (PMC4378510; doi:10.1038/srep09336)
Supplement: Supplementary Information [file srep09336-s1.docx]

***Lactobacillus rhamnosus* lowers zebrafish lipid content by changing gut microbiota and host transcription of genes involved in lipid metabolism**

Silvia Falcinelli^1^, Simona Picchietti^2^, Ana Rodiles^3^, Lina Cossignani^4^, Daniel L. Merrifield^3^, Anna Rita Taddei^5^, Francesca Maradonna^1^, Ike Olivotto^1^, Giorgia Gioacchini^1^ and Oliana Carnevali^1*^

**Supplemental Information**

**
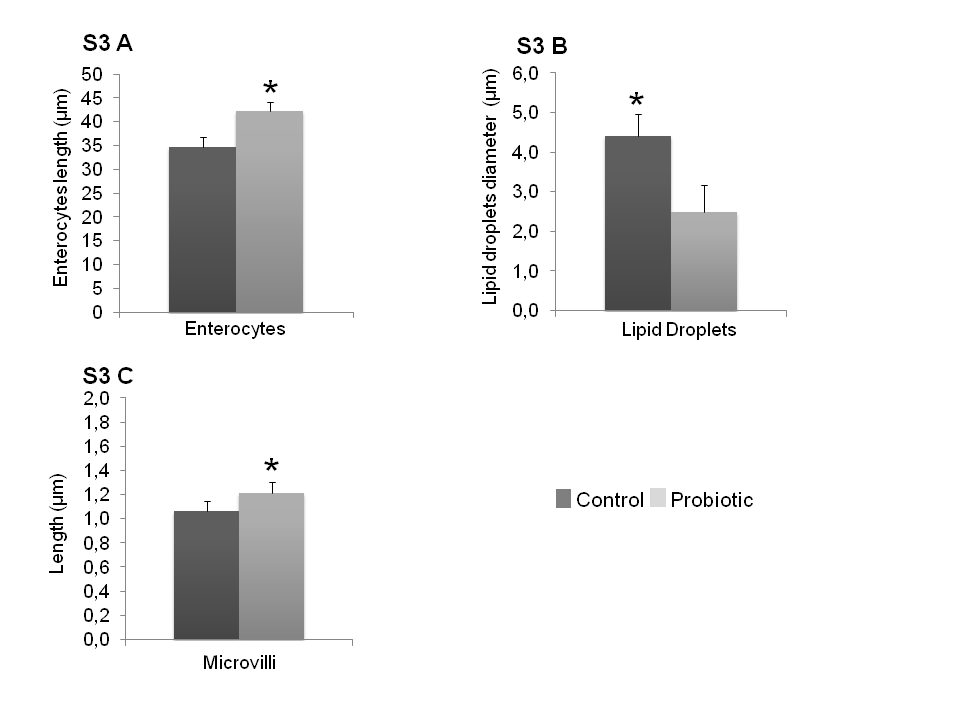
**

**
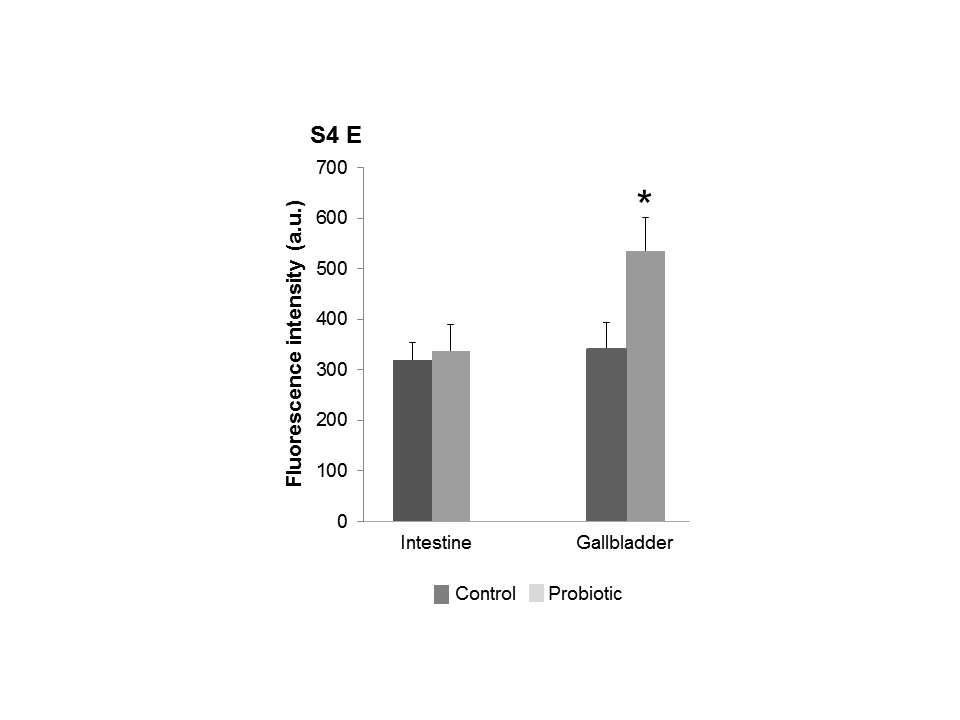
**

**FIGURES LEGEND**

**Figure S3.** **Measurement of intestine structures from Transmission Electron Microscopy (TEM) images shows higher enterocytes and microvilli length and smaller lipid droplets in the intestine of probiotic treated zebrafish**

From micrographs, enterocytes length of control and probiotic zebrafish larvae were measured. Results show significantly higher length of enterocytes in the probiotic treated larvae intestine compared to the control larvae (A). In probiotic treated larvae the size of the lipid droplets was significantly smaller compared to the control (B). From micrographs, microvilli length of control and probiotic zebrafish larvae were measured. Results evidence significantly higher length of microvilli in the probiotic treated larvae intestine (C). The significance of differences between groups for these values was determined using Student’s t-test. Data are the mean ± s.d. Asterisk indicates significant differences (P < 0.05).

**Figure S4 E.** **Quantification of fluorescence of intestine and gallbladder of control and treated zebrafish soaked in BODIPY 505/515.**

BODIPY 505/515 imaging show no differences of green fluorescence intensity in the intestine tract of both control and probiotic treated group in 6 dpf zebraﬁsh larvae. Probiotic treated larvae exhibit significantly enhancement of fluorescent signal in the gallbladder with respect to the control (P < 0.05) highlighting an accumulation of non-polar fatty acids. Quantification of ﬂuorescence expressed in a.u. The data are reported as mean ± s.d from 3 individual experiments. The significance of differences between groups for these values was determined using Student’s t-test. Data are the mean ± s.d. Asterisk indicates significant differences (P < 0.05).
